# Beyond Distinction: Private Art Museums and Their Versatile Role for Elites' (Self)Legitimization Discourses

**DOI:** 10.1111/1468-4446.70102

**Published:** 2026-03-11

**Authors:** Sara de Andrade Silva, Kristina Kolbe, Olav Velthuis

**Affiliations:** ^1^ University of Amsterdam Amsterdam the Netherlands; ^2^ Sociology of Arts and Culture Erasmus University Rotterdam Rotterdam the Netherlands; ^3^ Department Chair/Sociology of Economic Life & Culture University of Amsterdam Amsterdam the Netherlands

## Abstract

The 2000s have witnessed a significant, worldwide boom in new art museums founded by private, wealthy collectors. While the arts have long been a key arena for the remaking of elite distinction and the reproduction of inequalities, this surge in private museums has sparked much controversy. In this paper, we demonstrate how wealthy elites deploy this form of cultural philanthropy for (self)legitimation. Based on topic modelling analysis, we examine the online mission statements and ‘about us’ sections of 399 private museums across 59 countries to understand what forms of legitimation discourses they construct. We find that, beyond discourses of intra‐elite distinction, the mission statements additionally mobilize discursive legitimation strategies that highlight private museums and their founders as reliable, institutionalized agents in the artworld and valuable philanthropic actors in society more broadly. Overall, our analysis demonstrates how the arts function as a particularly versatile and powerful tool for symbolic elite legitimation struggles, allowing wealthy elites from different backgrounds to coalesce globally around private art museums. In light of escalating wealth concentration and widening economic disparities around the world, our paper adds to sociology's critical imperative to scrutinize the formation and reproduction of contemporary elites.

## 1 Introduction

Throughout history, the arts have served as a powerful site for elites to assert their status, influence, and wealth. While rulers, royalty, religious leaders, and aristocrats in particular preferred to associate themselves with the arts, they have long been accompanied by economic elites, too. Think, for instance, of the arts patronage of the Medici family in the Italian Renaissance or of the industrialists during the American Gilded Age as particularly prominent examples. These elites usually shared their collections with the public, to impress, educate, or discipline them (e.g., Dimaggio 1982; Higonnet 2009; Walker 2019). Especially from the 19th century onwards, economic elites have regularly converted their private collections into publicly accessible institutions such as the Frick Collection in New York or, in the 20th century, the Guggenheim Museums. The 21st century has brought a new, pronounced shift toward transforming art collections into private museums (Larry's List 2016; Velthuis et al. 2023), with several hundred of these institutions being established worldwide. Arguably, this boom reflects growing wealth concentration today, both in and beyond the artworld. It makes private art museums inextricably linked to the wider question of how current elites seek to legitimize their economic privilege and status.

While funding a private museum was, and still is, an undeniably expensive endeavour (Adam 2021; Larry’s List 2016), founders of these museums not only personally pay for them, but also frequently name these institutions after themselves and stay involved in their management, further indicating that private museum owners see themselves reflected in and represented by their institutions. Like other forms of (cultural) philanthropy, founding a private museum is frequently portrayed by elites themselves as a generous way of ‘sharing their passion’ or ‘giving back to society’. This discourse of giving and sharing seems to enable elites to legitimize their wealth and thus hold on to their economic class position (Kolbe 2023; Velthuis et al. 2023).

How private museums serve their founders' (self)legitimation strategies has so far not been studied in a systematic fashion. Some studies have explored the link between (private) museums and elite (self)legitimation in more depth (Ajana 2015; Kolbe 2023), yet these often only focus on one specific museum or country context and thus bear limited insight into private museums as a global phenomenon. Further, museum founders' acts of (self)legitimation through their museums have been riddled with notable controversies that have spanned various social domains. For instance, critics have seen private museums as a form of ‘art washing’ which should redirect attention away from dubious sources of the patrons' wealth (Shaked 2022; Spence 2024). Others have argued that philanthropic money should be spent on more urgent causes and have doubted the public benefits of private museums ‐ a critique that becomes especially salient in countries that offer tax benefits to museum founders based on the assumption that they would contribute to the wider societal good (Brown and Pes 2018; Boucher 2020; Robson 2019). And even within the artworld itself, the status and institutional legitimacy of private museums are often contested and their founders judged as trend‐adhering, vain, and ultimately market‐driven collectors (Brown 2019, 2020; Foster 2015). Given these different debates, one cannot take for granted that private museums generally help their founders signal legitimacy and, if so, how precisely the latter use their museums to channel discourses of (self)legitimation.

In this paper, we address these debates, aiming to provide a more systematic account of the role private museums occupy in elites' (self)legitimation work. To do so, and also to grasp the global scope of today's private museums, the paper draws on two key data sets. First, we established a global database of all private (contemporary) art museums to date (Velthuis et al. 2023). Second, we collected the mission statements published on the institutional websites of these museums. Widely used by organizations as a tool to emphasize their vision, uniqueness, and identity (Alegre et al. 2018), mission statements can also be used as a strategy for legitimacy (Suchman 1995).

Adopting a topic modelling approach, this paper takes a systematic look at the content of these statements. By mapping the different discursive narratives deployed by the mission statements, we aim to better understand the social role of these new private museums for the (self)legitimation strategies of today's economic elites. In doing so, we combine a (post)Bourdieusian lens with critical philanthropy literature in order to examine how these discourses of legitimation work, and what they can tell us about the relationship between economic elites and the arts today.

The paper proceeds as follows. First, we discuss the theoretical debates that underpin our interest in private museums as sites of elite (self)legitimation. Next, we introduce the museum database, detailing the mission statement sample and our topic modelling approach. We then move on to the analysis of the ten narratives the topic model identified. In the discussion and conclusion section, we take a critical look at what our findings mean for broader debates around elite legitimation and reproduction, both in the artworld as well as in society more widely.

## 2 Theory

Amidst mounting global wealth concentration and rising economic inequality, a pressing need exists for a rigorous analysis of how today's elites are formed and perpetuated (Cousin et al. 2018; Savage and Hjellbrekke 2021). Sociologists have approached this issue from various perspectives, studying, for example, the role of schooling for elite reproduction (Khan 2012; Reeves and Friedman 2024; Worth et al. 2023), elite's consumption and leisure practices (Kolbe 2023; Lena 2019; Mears 2020; Sherman 2018) or, notably, their philanthropic engagements (Maclean et al. 2021; Maclean et al. 2021; McGoey 2012; Reich 2018). This burgeoning literature is inspired, among others, by the observation that, while economic research has elucidated how the affluent have incrementally widened their share of income and wealth, scant attention has been paid to the cultural mechanisms and moral discourses that support these processes and that enable elite reproduction (e.g., Lamont et al. 2014). A current concern in the study of elites is therefore to develop a better understanding of the discursive approaches used by elites themselves to achieve, or at least *to signal* their own legitimacy as elite actors (Kantola and Kuusela 2019; Kolbe 2023; Maclean et al. 2021). Importantly, this concern comes to the fore in an even stronger manner today given that economic elites, as they consolidate further and seem to stake out more power across different social and political areas, arguably also face increasing attention and scrutiny directed at their resources and influence (Maclean et al. 2012).

Elites have a wide range of tools and strategies at their disposal to signal legitimacy for themselves and their riches, such as Corporate Social Responsibility programs in their firms, hiring PR agents who control their image in the (social) media, or engaging in various forms of philanthropy, including in the field of culture. In this paper, we specifically explore how private art museums, as central sites of contemporary elite philanthropy in the arts, might play a role in such (self)legitimation work (see also Bourdieu 1985; Dimaggio 1982; Accominotti et al. 2018; Lena 2019). Dating back to the Gilded Age, private museums have a history of serving their founders as vehicles for transforming economic into symbolic capital. Their museums helped businessmen like Whitney, Guggenheim, or Frick gain social status and prestige; the association with the arts assured that they were enshrined as legitimate elites even long after their own passing (e.g., Dimaggio 1982; Higonnet 1997).

Simply put, this paper scrutinizes whether and how their contemporary equivalents seek to do the same. Here, we build on a recent study by Kolbe (2023), who found that private art museums indeed offer their founders ‘productive discursive sites’ (Kolbe 2023, 3) through which to formulate narratives of (self)legitimization as both effective and innovative agents in the artworld and as responsible, worthy, and ethical elite actors more generally. Kolbe's findings are mirrored by other research on private museums that notes how founders cast their projects in terms of ‘sharing their passion for art with the public’ or indeed ‘giving their wealth back to society’ (e.g., Walker 2019). However, none of these studies have approached private museums on a global scale but have instead favoured in‐depth studies of specific cases or country contexts over a more systematic analysis.

The question of whether today's private art museums can serve their founders as vectors of (self)legitimization is, moreover, compounded by the fact that many heated debates surround these institutions. In the art field, critics cast many founders as wealthy but culturally thin, who follow market trends and dealer advice instead of building their collections in an autonomous and disinterested manner (Brown 2019, 2020). What's more, bodies like the International Council of Museums (ICOM) exclude private art museums as members and recently, have pointed them out as a potentially dangerous form of competition for (financial) resources in the field (IRAPFM 2025). Private museums are therefore often criticized as fleeting institutions and mere vanity projects of the super‐rich, who are more interested in keeping pace with global elite leisure trends than demonstrating a genuine, long‐term commitment to art (Dercon 2017).

Private museums have also been scrutinized as philanthropic endeavours more generally, especially in view of the tax benefits some countries grant them (e.g., Brown and Pes 2018; Robson 2019). Major philanthropists such as billionaire Bill Gates have probed whether the resources spent on the arts would not better serve other social needs.^1^ Others have judged private museums as particularly wasteful displays of wealth rather than as sites of wealth legitimation. The Crystal Bridges museum, established in 2011 by Alice Walton, the billionaire heir of the family which established the American supermarket chain Wal‐Mart, is a case in point. Its opening sparked controversy as the company was long known for its poor labour standards (Helmore 2011). At many other occasions, founders have been lambasted for the origins of their wealth, like the Russian oligarch and Putin‐friend Roman Abramovic (Rushing 2022), who founded a private museum in Moscow, or German collector Julia Stoschek, whose art foundation is funded with family wealth accumulated through forced labour during the Third Reich (Rogers 2022). And even within elite circles, it no longer seems self‐evident that the arts still function as a refined form of distinction—with a private museum functioning like a moralized alternative to luxury yachts or private jets. While some founders seem to use museums to signal ‘good’ wealth (Kolbe 2023), arts philanthropy increasingly reads as snobbish (Jung 2015; Milner and Hartnell 2015) and elites' desire to appear ordinary (Bryson 1996; Peterson and Kern 1996; Prieur and Savage 2013; Farrell 2020; Reeves and Friedman 2024) can blunt cultural signalling. Lastly, the high costs of running a museum, paired with evidence that many of them close after a founder's death (Velthuis and Gera 2024), suggest heirs may prefer preserving wealth, which means that legitimization claims are moreover contingent on peer and familial acceptance.

In light of these controversies, we therefore assume that the effectiveness of private museums as tools for elite (self)legitimation cannot be taken for granted. Instead, this legitimacy needs to be discursively constructed by elites themselves, and we turn toward museums' mission statements to explore exactly how. To that end, we combine a (post)Bourdieusian perspective (Bourdieu 1984, 1993) on elite distinction and elite philanthropy literature (e.g., Heemskerk and Takes 2015; Maclean et al. 2021; Saunders‐Hastings 2024; Schmitz et al. 2021; Toepler 2022) to see whether mission statements may position institutions and their founders as legitimate elite agents, not just in the artworld but perhaps also vis‐à‐vis other elite groups or even society more widely. Our analysis thus bears insights not only for current research on the arts and private museums specifically, but also contributes to wider sociological debates on how contemporary economic elites signal legitimacy for their wealth, status, and power.

## 3 Data and Methods

The paper draws on an exhaustive database of private art museums and their founders worldwide, established at the University of Amsterdam using publicly available sources, systematic web searches, and combining pre‐existing databases (for the data collection methodology, see Velthuis et al. 2023). We define private art museums as founded and controlled by one or several private persons, which receive no or limited public funding, have a permanent collection of modern and/or contemporary art, and make this collection accessible to the public in a physical structure on an ongoing basis (Velthuis et al. 2023). With corporate museums excluded, the database thus centres on private individuals, reflecting a specific segment of today's economic elite with the resources to collect art and create and financially sustain such institutions based on their own private collections. It is noteworthy that even within this seemingly narrow definition, the private museums captured in the database differ significantly in terms of size, ambition, and level of professionality: they range from art collections shown in private residences by appointment only, over small‐scale ventures with one or two core staff and limited public access, to large, fully‐fledged institutions open most days that closely resemble public museums—with valuable collections, rotating exhibitions, curatorial and conservation teams, educational programming, cafés, and museum shops.

Overall, the database includes 446 private museums for modern and contemporary art across 61 countries (See Table 1). Over 80% of them opened their doors in the new millennium (see Table 2), reflecting rapidly increasing wealth inequality worldwide. Most of the museums are located in Germany (60 museums) and the United States (59 museums), but countries like South Korea (50 museums) or China (28 museums) host many as well, while the number has been growing from 3 in 2003 to 18 in 2022 in Latin America (including institutions which have been widely discussed in the international press such as Inhotim in Brumadinho, Brazil, or Jumex in Mexico City) and from 1 to 8 in Africa in the same period. For this paper, we analysed 399 of these museums across 59 countries, focusing on those for which mission statements were available (see Table 1).

**TABLE 1:** Top 15 private museum countries.

Country	Number (percentage)
United States	57 (14.3%)
Germany	56 (14.0%)
South Korea	47 (11.8%)
Italy	23 (5.8%)
China	20 (5.0%)
Spain	20 (5.0%)
France	15 (3.8%)
Belgium	11 (2.8%)
Switzerland	10 (2.5%)
United Kingdom	9 (2.3%)
Greece	9 (2.3%)
Japan	8 (2.0%)
Turkey	7 (1.8%)
Brazil	7 (1.8%)
Russia	7 (1.8%)

**TABLE 2:** Key statistics of private museums and their founders.

	*N*	Minimum	Maximum	Mean	Std. deviation
Age of founder at opening of museum	258	24	93	57.88	14.433
Opening year museum	392	1960	2022	2006.96	11.065
Collection size (number of artworks)	177	9	18,300	1604.22	2906.936
Wealth of founder (in million USD)	64	4	184,500	13038.66	28459.677

The database contains information on 405 museum founders (some of them founded more than one museum). Most are men (57.9%) or couples (26.6%), while only 15,5% were founded by women. Their average age at the moment of founding is 58. Using publicly available sources, we managed to retrieve the wealth of 58 individuals, who founded 75 museums in total (some founded more than one museum). On average, the personal wealth of members of that group amounts to 10.8 billion USD. Just as strikingly, 37 individuals, or almost 10% of the founders included in our database appear or have appeared in Forbes' annual rich list with a wealth of at least 1 billion USD. Among them are the French luxury goods tycoon Bernard Arnault, Walmart‐heir Alice Walton, and the currently sanctioned Russian oligarch Roman Abramovich. 88 founders (21.7%) have at least once appeared on the list of Top 200 most important art collectors worldwide, which is annually published by the American arts magazine ARTnews.

**FIGURE 1:**
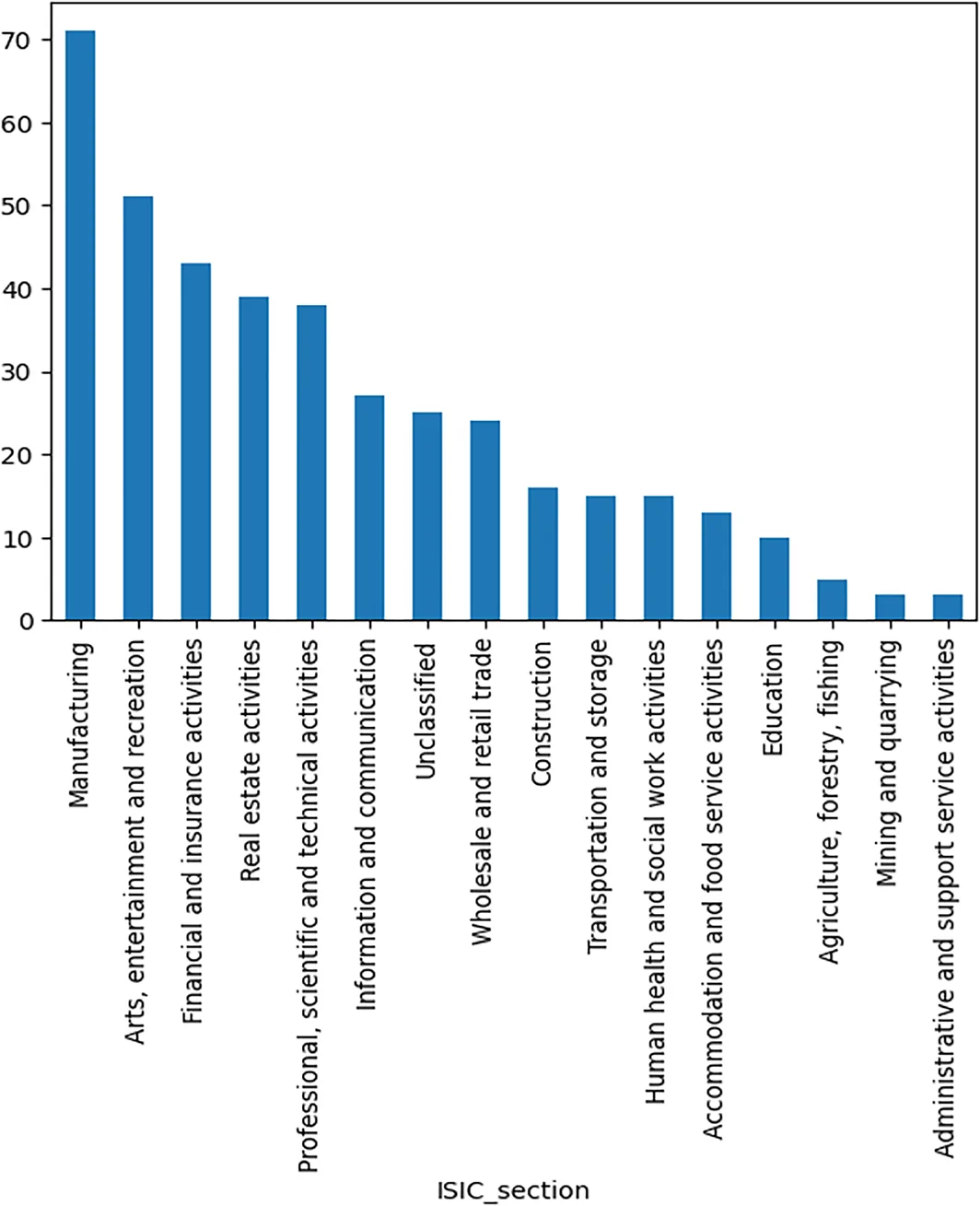
Industry Background of museum founder.

The private museum founders in our database made their fortunes across different industries.^2^ The greatest proportion of them (60 or 21.5% out of the 279 founders for which industry data could be collected) is or was active in manufacturing, followed by arts, entertainment and recreation (18.6%), the financial sector (12.9%), and real estate (12.5%; see Figure 1).

The majority of founders are ‘self‐made’ men and women, who amassed their wealth as entrepreneurs. We identified only 18 founders who relied wholly or partially on inherited wealth or ‘old’ money.^3^ People working in the public sector are conspicuously absent, which makes sense considering the salary levels and the high costs of collecting art, establishing a museum, and financially sustaining it.

A strong indicator of the conflation of the founder and their museum is the fact that 48% of all institutions in our database carry the name of their founder. This is further reflected in the mission statements, which we collected in January and February 2024 from the respective museums' websites: while we do not assume that these statements are always written by the founders themselves, they tend to be either signed in their name, or do almost invariably mention the founder, often repeatedly, for instance by recounting how they started collecting art, what his relationship is to art, or what his vision is for the museum. In that respect, we see mission statements as one of the crucial discursive spaces for this specific elite group to position and legitimate themselves vis‐à‐vis other members of the wealthy elite, the artworld, and wider publics (e.g., Castelló et al. 2016; Chauvin, Cousin and Mears, forthcoming, De Keere and Burchartz 2025; Hearn and Banet‐Weiser 2020; Larsen 2017). If no explicit mission statement was available on museums' websites, we sought to retain all information related to the museums' founding from other sections of the website, such as the ‘about us’ section. After filtering out museums without a mission statement or comparable textual content on the museum's website, our final sample comprised 399 cases across 59 countries.

In order to identify underlying themes and their prevalence occurring across the mission statements, we adopted a topic modelling approach (Egger 2022). For this analysis, we used a Nonnegative Matrix Factorization (NMF) model (Chen et al. 2019; Egger 2022; Egger and Yu 2022). Based on the coherence scores of the topic model (Röder et al. 2015) and the interpretability of the outcome, we selected a 10‐topic model. Based on their 20 most relevant keywords and on a close reading of the 10 mission statements that scored highest on each topic, we labelled each of them (see Table 3) and interpreted them in terms of distinct narratives which we will discuss and cluster in the next section.^4^ We also conducted a correlation analysis linking the scores (which express the degree to which each topic is present in a mission statement) with key characteristics of founders (such as gender; age; industry background; top collector according to ARTNews) and their museums (geographic location; social media presence; whether they carry their founders' name; focus on contemporary or modern art) in an attempt to understand possible patterns between the use of (self)legitimation narratives and these characteristics.

**TABLE 3:** Narratives and top 20 words related to each of them.

Narrative	Top 20 words
Narrative 1: Institutionalizing the foundation	Foundation; art foundation; public; established; program; support; institution; mission; nonprofit; state; knowledge; founder; artistic; institute; project; initiative; research; young; founded; education
Narrative 2: Crafting a museum identity	Museum; art museum; museum art; opened; modern; visitor; world; building; design; founder; educational; main; public; research; director; permanent; hall; contemporary art; abroad; held
Narrative 3: Origins of the founder's interest in art	Painting; year; collector; family; sculpture; began; passion; collecting; art collection; early; including; private; university; interest; century; many; time; american; well; school
Narrative 4: Supporting contemporary art	Contemporary art; international; public; support; space; project; private; curator; program; audience; collector; founded; platform; production; exhibition space; practice; creation; focus; artistic; centre
Narrative 5: The founder's life with art	My; world; business; time; life; always; love; across; people; it; making; day; make; mean; collector; way; founder; accessible; present; practice
Narrative 6: Foregrounding museum architecture	Nature; space; building; house; architecture; place; site; opening; project; experience; architect; designed; area; unique; visitor; garden; park; landscape; time; artwork
Narrative 7: Exhibiting art in gallery spaces	Gallery; space; building; architect; year; became; show; exhibition; space; permanent; series; opened; construction; american; contemporary art; feature; including; designed; organization; includes; important
Narrative 8: Social commitments	Social; culture; cultural; commitment; group; institution; artistic; promote; society; well; project; company; activity; way; creation; business; year; development; creative; idea
Narrative 9: Dedication to the artistic medium	Photography; medium; publication; dedicated; collecting; research; history; aim; understanding; field; historical; international; opened; focus; original; video; program; installation; architectural; dialogue
Narrative 10: Serving the local community	Art museum; cultural; local; culture; museum art; various; school; program; space; community; experience; education; region; art culture; artistic; people; life; centre; creative; established

## 4 Findings

The topic model generates three key findings: first of all, it demonstrates that mission statements do not depend on one narrative exclusively. As if the founders are hedging their bets in playing the legitimation game, most of their mission statements are an amalgam of several narratives. Secondly, while we had expected that these museums would be showcases for the global elites' refined artistic taste, we find a range of strategies which go beyond such a Bourdieusian logic of distinction. In order to discern the discursive (self)legitimation work of mission statements in more detail, we have grouped the 10 narratives into three key discursive clusters, which speak to (a) intra‐elite distinction struggles, (b) legitimation concerns in the artworld, and (c) the role of museums and founders in society more broadly. We will discuss these three clusters in more depth in the remainder of this section. Our third finding, which we will turn to in the next section, is a remarkable convergence in mission statements, with few systematic differences in the narrative strategies of founders when it comes to for example gender, industry background, or ‐ with a few exceptions ‐ region.

### 4.1 Intra‐Elite Distinction

Unsurprisingly, the mission statements of private museums signal status toward other elite groups. We find that a range of narrative strategies is deployed to that purpose. First of all, one narrative (no. 3; see Table 3) highlights the origins of the founder's interest in art and trajectory of art collecting. Opposing imaginaries of art collecting as driven by short‐lived fashions among elites, it emphasizes how a personal engagement of art developed early on in the collector's life and became a defining characteristic of his biography. Specifically, as part of this strategy, the founder's distinguished taste and embeddedness in the artworld are highlighted and museums are often cast as the emotional apex of their lifelong romance with art, one that they now seek to share with broader audiences. Or, to put it in the words of the US‐based Powers Art Centre's mission statement: ‘collecting brought tremendous joy to [founder] John and Kimiko's lives, but John was especially passionate about sharing their love and appreciation for contemporary art […] a wonderful way to connect with others and prompt meaningful conversations.’ Distinction and legitimacy are here signalled by portraying museum founders as disinterested, genuine, and truly embedded agents in the artworld, and thus as distinguished and autonomous elites more broadly (e.g., Bourdieu 1993; Braden 2016; Kolbe 2023; Moulin 1987).

A second, interrelated narrative (no. 5) further details how the founder's life is intertwined with the arts. Mission statements that endorse this narrative tend to be written in the name of the museum founder, often in the first person, and include an inspirational quote by them. This narrative presents art patronage as a profound personal passion of the founder, which is frequently shared with the founder's partner or runs deeper in the family. These mission statements may also recount in detail how the founder's collecting practice is interwoven with their business successes or personal travels. The Germany‐based Museum Barberini, for example, introduces billionaire entrepreneur Hasso Plattner not only as the museum's ‘founder and benefactor’ but also as a prominent international business figure: ‘the cofounder and longtime chairman of the software company SAP, Prof. Dr. h.c. mult. Hasso Plattner is one of Germany's most prominent entrepreneurs, whose name precedes him throughout the world. […] With an impeccable eye for quality, Hasso Plattner has amassed one of the most significant collections of Impressionist and Post‐Impressionist works over the past 2 decades’.

By emphasizing founders' entrepreneurial successes and, indeed, even drawing a line between their strong position in the business world and their noteworthy position in the arts, this discursive strategy is a clear way for founders to showcase both their economic and cultural capital while also demonstrating their overall personal status. Arguably, this shows how private museums offer economic elites a tool to draw symbolic boundaries between themselves and other elite actors who may have similar wealth at their disposal yet may lack founders' cultural knowledge and prestige. Signalling cultural capital in these ways might be especially important for this elite group, who ‐ according to our database ‐ are more often self‐made entrepreneurs rather than ‘old money’ heirs and may therefore face greater pressure to symbolically legitimize their elite status. These findings tie in with other research on intra‐elite legitimation struggles through private art museums. Kolbe (2023, 1121), for example, finds that the museum founders in her study aim to construct intra‐group status hierarchies by presenting themselves as authentic, publicly minded, and ultimately ethical elite members ‘who ought to be distinguished from more money‐driven, ostentatious, and ultimately unethical private elite actors ‐ both in the artworld and beyond’.

This may be a particularly potent strategy of (self)legitimation for founders, given that within elite circles, the status benefits of arts philanthropy are not a given, while economic elites more broadly might be less concerned with signalling their cultural capital overall. Philanthropic donations to the arts are increasingly seen as a sign of snobbery and judged for their vague social outcome (e.g., Milner and Hartnell 2015; Jung 2015). This holds particularly true in light of the rise of the cultural omnivore (e.g., Peterson and Kern 1996; Prieur and Savage 2013; Bryson 1996) and the desire of elites, especially in countries like the United States (Farrell 2020) or the United Kingdom (e.g., Reeves and Friedman 2024), to come across as ordinary. However, we actually find that private museum founders use their engagement with art to craft a rather distinguished, culturally knowledgeable image of themselves: one that we argue serves specifically as a sign of distinction toward other economic elite groups by signalling both cultural and even moral status (e.g., Bourdieu 1984; Kolbe 2023; Sherman 2018).

Here, we find yet another discursive way in which founders signal status; namely, by mentioning the consecrated architects responsible for the design or reconstruction of their museum edifices, many of whom are affiliated with esteemed architectural firms such as Frank Gehry, Tadao Ando, or Renzo Piano. In doing so, they straddle a fine line between demonstrating good taste and engaging in conspicuous consumption: while branding this ‘extreme iconicity’ of the museum building might present a pathway for founders to create a parallel between their museums and other celebrated institutions, it also poses a risk for (self)legitimation as they might be interpreted as signs of conspicuous consumption rather than displays of good architectural taste and commitment with supporting artistic production. The art historian and critic Clair Bishop, for instance, argues that it has made ‘the museum's external wrapper […] more important than its contents’, leaving the art they exhibit either ‘lost inside gigantic post‐industrial hangars’ or ‘supersizing to compete with its envelope’ (Bishop and Perjovschi 2014). At the same time, however, this narrative offers an opportunity for intra‐elite legitimacy as it demonstrates at once the financial resilience to follow through with such a high‐, cost initiative *and* the cultural capital to recognize and hire the right museum architects. Beyond the signalling of status alignment with the fields of cultural production, this discursive positioning can also be seen as a response to a sustained critique which has been levelled against private museum founders within the media, the arts, and academia. According to such critics, private museum founders lack sophisticated taste, are ego‐driven, and are prone to following market trends (e.g., Adam 2021; Bechtler and Imhof 2018) ‐ countering such an image may therefore be a key concern in founders' wider legitimation struggles.

### 4.2 Legitimation Concerns in the Artworld

With regard to other aspects of how private museum founders seek to construct their legitimacy as worthy cultural actors through the positioning of their museums, a Bourdieusian perspective is less apparent or useful. This holds in particular for the narratives (no.1 and 2, Table 3) which are centred around the institutionalization of the foundation and a specific museum identity. The foundation narrative highlights the non‐profit character as a milestone for private collections turning public. Statements often contain a timeline of the institutions' development, indicating their increasing institutionalization over time. They also tend to reinscribe philanthropic discourses of ‘giving back’ or supporting the arts and society more widely through their foundation, thus legitimizing the accumulation of wealth. Likewise, mission statements which endorse the ‘crafting a museum identity’ (no.2, Table 3) narrative want to drive down the point that they are not just private exhibition spaces, but museums proper, including an exhibition program, an aesthetic focus, a permanent space which is open to the public on an almost daily basis, as well as research and educational programmes.

These narratives signal that the founders' engagement with art is a serious, professional, and grounded endeavour rooted in established practices. In this vein, they tend to include discussions of museums' organizational histories and present changes in governance or policy as crucial steps, further reinforcing their institutional legitimacy. As such, they resonate with wider, long‐term developments within the museum sector, in which public agencies and governments demand transparency and accountability as evidence of good management (Harth and George 2025; Mendoza and Talavera 2025; Pabst 2016). We interpret this emphasis on organizational aspects as a way of claiming legitimacy by bringing private museums and their founders closer to their already established (public) counterparts and by copying their standardized working practices (e.g., DiMaggio 1991). Such a discursive logic seems to furthermore reflect broader discourses around private entrepreneurialism and patronage, which affirm elite's philanthropic engagements as valuable and durable contributions to public (art) life. Founder's private economic and symbolic accumulation is here framed as serving a collective benefit, normalizing ‐ and indeed helping to legitimize ‐ the expanding influence of private elites across the cultural sphere (Maclean et al. 2012; Maclean et al. 2021; McGoey 2012).

Against this backdrop, we identified yet another discursive strategy which signals the desire of founders to be seen as full‐fledged players in the arts and in the field of contemporary art specifically. This ‘supporting contemporary art’ narrative (no.4, Table 3) positions the museum as an active participant within a broader discourse of the avant‐garde, dedicated to the production of knowledge of a specific artistic media, and as a supporter of the emerging arts, thereby situating itself as a relevant and globally oriented actor within the contemporary art scene. Central to this narrative is an emphasis on curatorial work, the museum's commitment to innovation, and artistic research. Mission statements with a clear focus on contemporary art explicitly champion the ‘support of creation, production, and dissemination’ of contemporary art; ‘explor[ing] the most potential young artists,’ as Guangdong Contemporary Art Centre in China claims; or connecting artistic production with social causes, such as the Girls' Club Collection, which focuses on ‘nurtur[ing] the careers of female artists’. With a focus on residency programs and commitment to young artists, these museums promote an image of artistic relevance for themselves and their founders. Similarly, museums, whose mission statements endorse the related narrative of commitment to the artistic medium (no.9, Table 3), demonstrate interest in scholarly research and conservation of its archive, which mostly consist of photographical works.

Overall, this emphasis on meeting institutional standards and lending meaningful support to the arts implies more than simply a founders' personal interests in the field. Rather, such a discursive strategy presents founders as serious and embedded actors in the field, who strive to build durable, serious, and lasting museum spaces. In doing so, it addresses concrete concerns which have been voiced in the artworld against private museums ‐ and against philanthropic projects of elites more generally ‐ that they would be whimsical, fleeting, and therefore short‐lived; vanity projects of the super‐rich who do not want to be seen left behind in a global fashion rather than the expression of a serious and lasting commitment to art (Dercon 2017). As former MoMA head curator Robert Storr summarizes it: ‘It's hard to name a great collector who became a great museum director, even of their own museums’. The International Council of Museums (ICOM) even argues that these private institutions should not qualify as ‘museums’ to begin with (IRAPFM 2025) ‐ a position which we see actively countered in mission statements.

### 4.3 Role of Founders in Society

Finally, a third set of narratives emphasizes that private museums are more than status‐signalling projects and that mission statements do more than merely respond to artworld‐specific critiques. Notably, they also seem to respond to broader concerns that in founding private museums, wealthy elites fail to create genuine philanthropic and social value. This concern may arguably tie in with broader, more general critiques against private philanthropy; namely, that the latter would provide today's wealth elites with the opportunity to portray themselves as publicly facing, socially engaged, and ultimately moral elites who use their money in a redistributive manner (e.g., Kolbe 2023; Walker 2019).

We identify two interrelated narratives through which this issue is addressed across our mission statement sample. The first (no.8, Table 3) underlines the founder as a socially responsible actor, who is committed to improving social welfare and develops a social investment strategy. This narrative depicts private museums not as exclusive or even as elite spaces, but as public‐facing. Corresponding mission statements tend to mention their program of inclusive, accessible events, or workshops, thereby positioning the museums ‐ and by extension, their founders ‐ as serious, engaged contributors to public arts provision and education. The mission statement of the Fundación Marcelino Botín, for example, depicts the museum as ‘an engine for generating economic, social, and cultural wealth on the Cantabrian coast’ and underlines that it ‘was created in 1964 by Marcelino Botín Sanz de Sautuola and his wife, Carmen Yllera, to promote social development in Cantabria […] contributing to the overall development of society by exploring new ways to uncover and support creative talent.” The Fundación organises programmes in the realms of the arts and culture, education, science and rural development, and supports social institutions in Cantabria so as to reach those who need it the most.’

Another clear illustration is offered by Borusan Contemporary in Turkey, whose mission statement ‐ written in the name of its founder Ahmet Kocabıy ‐ acknowledges that ‘one of our priorities has always been to return to this country what we have gained from it. […] Today, one of the main axes of the social responsibility activities of Borusan, hosted under the Borusan Kocabıyık Foundation, is arts and culture.’ Overall, this narrative appears to legitimize the elite status of private museum founders through accentuating a wider (self)entrepreneurial lens that echoes a broader and more familiar story often championed by billionaires like Bill Gates or even Elon Musk. In this worldview, immense wealth is justified by the belief that the same private, entrepreneurial drive that fuels business success can also be harnessed to tackle social problems ‐ often more effectively than governments can (e.g., McGoey 2012; Reich 2018).

Second, the civic legitimacy for founders and their museums is moreover constructed by stressing how the institution engages with local communities (no. 10, Table 3). This strategy underscores museums' public‐facing, socially engaged nature in an even more explicit manner. Bringing together terms such as ‘local’, ‘culture’, ‘community’, ‘space’, ‘experience’, ‘education’, and ‘region’, this cluster specifically shows how private museum founders wish to position their institutions as open and accessible spaces for community activity and for local cultural development. It is noteworthy that a number of mission statements that score high on this narrative seem to place a particular focus on their active and comprehensive engagement with local audiences, either stressing how they would bring international art to their local community or how they would care for local arts and cultural heritage. The Woojong Museum in Japan's Namdo region presents a striking example in this context, specifying that its mission is ‘to revitalize the development of local art by reflecting on and encouraging the artistic orientation of Namdo and spreading the centralized cultural and artistic experience to the region. It is the responsibility and duty of the museum to provide win‐win and cultural services with the local people.’ On the whole, such a legitimation strategy plays into wider discourses around elite philanthropy through which wealthy private individuals might be able to reject the image of the self‐centred, ostentatious, and money‐driven elite and instead represent themselves as moral and ethical agents who indeed serve a public good (Maclean et al. 2021).

These two interrelated narratives cannot, however, solely be reduced to strategic ways of deflecting attention from the founders' economic resources or legitimizing this wealth by ‘giving back to society’. Rather, these narratives also appear to reflect wider changes in the contemporary artworld, in which artistic research (Borgdorff 2010), community involvement (Bourriaud 2002), and participatory art (Bishop 2012) have come to compete with traditional avant‐garde values such as originality and artistic innovation. Yet, even when read in this light, it is noteworthy how these narratives repeatedly centre the civic worth of private museums and the wider philanthropic agency of their founders.

### 4.4 Global Convergence

Beyond analysing how mission statements construct discourses of (self)legitimation for founders and their museums, we also sought to develop a more fine‐grained understanding of how these different (self)legitimation narratives may be patterned across our global museum sample. As the museums in our database differ quite considerably in terms of their size, location, and founder characteristics such as gender, wealth and industry background, we expected to find insightful relationships between specific mission statements and the kinds of (self)legitimation discourses they produce ‐ reflecting, for instance, local cultural repertoires (Lamont and Thevenot 2000), or, founders with a specific industry background, for example in finance, manufacturing, or real estate, being more inclined to engage in conspicuous forms of cultural display. Via correlation analysis and One‐way ANOVAs we did find a limited number of statistically significant differences; for instance, older museum founders are more likely to craft a mission statement around the way their interest in art originated (narrative 3), while private museums located in Asia were more likely to emphasize the proper museum‐status in their mission statements (narrative 2). Interpreting each of these relations is beyond the scope of this paper, in particular because they do not seem relevant for the self‐legitimation strategies which are the focus of this paper. Moreover, while some characteristics did, most characteristics did not relate significantly to the various narratives. Indeed, what stood out was the global convergence in (self)legitimation strategies across our sample, with the 10 narratives functioning like a general repertoire which all founders draw from, by and large irrespective of their gender, wealth industry background, or status within the artworld and of the location, genre focus, or naming of their museum.

We understand this convergence of legitimation strategies in three ways: first of all, it resonates with the notion of a transnational global elite habitus (Rothkopf 2008; Chauvin and Cousin forthcoming) and suggests that today's art elites are not only interconnected through international markets and social networks but also through similar consumption patterns, forms of leisure life and parallel discourses of cultural authenticity, status distinction, and even civic virtue. We do not, however, claim that elites are truly de‐localized; rather, we propose that it is precisely the heterogeneity of (self)legitimatizing narratives mobilized by mission statements that enables elites of various backgrounds and regions to cluster globally around private art museums. Secondly, and more specifically, this convergence can be seen as part of the emergence of a global contemporary art field (Buchholz 2022), in which rules and conventions are at least to some extent globally shared by artists, curators, critics, collectors, and indeed museum founders, who run into each other at international art fairs, openings of museum shows, and similar events. Finally, and relatedly, we see this convergence as the manifestation of isomorphism within this field (DiMaggio and Powell 1983). The fact that the mission statements in our sample employ various (self)legitimizing narratives at once, yet display no discernible pattern, may suggest that today's globalized artworld revolves around similar discourses and values, and that contemporary museums have become particularly conformist institutions, whose museum designs, envisioned audiences, artistic programs, ways of exhibiting art and, indeed, mission statements converge.

## 5 Discussion and Conclusion

In this paper, we explored how private art museums play crucial roles in contemporary processes of elite‐making by serving their founders as constructive discursive sites of (self)legitimation narratives. Taking the rising numbers of private art museums from the 2000s onwards as our point of departure, we studied how mission statements allow elites to legitimate both their own status as well as their lavish spending on a museum of their own. While parallels can be drawn between today's global museum boom and the Gilded Age in the United States, when rapid economic expansion encouraged the creation of private museums, contemporary differences in cultural and material conditions profoundly shape how such initiatives are perceived, received, and justified. As DiMaggio (1991) reminds us, American museums historically emerged as educational institutions aimed at refining national taste, while claiming status for the Boston elites who institutionalized high culture. Unlike many European countries, where arts support relied heavily on the state, 19th‐century U.S. arts were largely considered a private affair (Zolberg 2000), and early museums ‐ quasi‐private clubs sponsored by local elites ‐ publicly professed the intention to ‘elevate the taste of a larger segment of the population,’ thereby legitimating any public funding and fulfilling what Zolberg (1984, 398) describes as a ‘newly perceived social obligation of spreading high culture to the masses.’

By contrast, today's boom unfolds within a densely populated, highly institutionalized, and professionally managed global art ecosystem. It moreover takes place against a backdrop of unprecedented wealth concentration with the top 1% amassing ever‐larger shares of assets (Piketty 2014, 2021). Elites now arguably face intensified moral and public pressures to justify their privilege, and private museums may offer strategic vehicles for founders to legitimize themselves both within the artworld and more broadly. Indeed, our analysis of 399 museums' mission statements across 59 countries shows how legitimation strategies for private museums themselves can also be read as discursive and arguably even moral repertoires of elite (self)legitimation. Using a topic modelling approach, followed by closely reading the corresponding mission statements, we identified 10 such legitimizing narratives. While some narratives appear to seek legitimacy for their founders by portraying themselves and their institutions as valuable, long‐standing agents in the art field, others mobilize broader discourses around elite distinction or private philanthropy, thereby signalling founders' legitimacy within wider elite circles and even society more broadly.

These narratives are spread across our mission statement sample in a rather unpatterned manner. Indeed, we found that mission statements of private museums are rather messy texts that frequently make use of various legitimizing narratives at once and without any apparent reason for prioritizing some narratives over others. We found few meaningful correlations between the use of a specific mission statement narrative and other characteristics of a museum or its founder. Instead, mission statements display a striking global convergence, drawing on a shared repertoire of legitimation strategies largely independent of founders' backgrounds, museum types, or locations. We interpret this convergence as evidence of a transnational elite habitus (Rothkopf 2008, Chauvin, Cousin and Mears, forthcoming) and a globalized contemporary art field (Bishop 2025; Borgdorff 2006; Buchholz 2022) characterized by isomorphism, in which private museums articulate remarkably similar discourses of cultural value, status, and civic purpose.

Partly, then, our analysis extends Bourdieu (1985),  (1993) insights on elite capital accumulation by underscoring how art museums offer wealth elites a potent vehicle for translating economic into symbolic capital: rather than relying on overt displays of wealth, founders emphasize disinterested connoisseurship, curatorial innovation, and a philanthropic ethos to signal legitimate elite status. This allows them to distance themselves from the commercial, and potentially ethically fraught, origins of their fortunes, while frequently still emphasizing their success as business entrepreneurs. This finding corresponds to Kolbe’s (2023, 1121) analysis of private museums as allowing their founders to effectively ‘blur the line between […] seemingly conflicting discourses of a market‐based private entrepreneurialism and artistic authenticity’, thus claiming legitimacy as worthy private elites across different cultural and social realms. We suggest that these discursive strategies aim to situate founders in the lineage of revered collectors like Frick, Guggenheim, Barnes, Whitney, or Gardner, whose legacies of philanthropy supersedes even their commercial success ‐ reiterating art's unique power as a status investment and a vehicle for enshrining elite legacy (Higonnet 2009).

Yet, beyond showing how private museums and their founders seek to position themselves as legitimate cultural players, this paper also contributes to renewed debates on cultural philanthropy by documenting how museum mission statements cast founders as engines of community development, research, education, and even as effective actors in tackling wider societal problems (Reich 2018; Maclean et al. 2021). In doing so, founders draw on public‐sphere discourses that de‐emphasize information about their wealth and museum financing (including its scope and potential tax breaks for the museum) and instead highlight privately driven social innovation and community development, mirroring Lamont et al. ’s (2014) argument that moral repertoires are central to elite legitimation in contemporary neoliberal contexts. We hold that this focus on civic‐oriented narratives both anticipates and responds to intensified scrutiny of elite giving (McGoey 2012; Reeves and Friedman 2024), both in the cultural sector and more generally.

Our findings furthermore echo current research on intra‐elite distinction. Notably, our overall analysis links to Friedman and Reeves’s (2020) observation that today's elites carefully curate their public personae to foreground cultural over economic credentials. However, while Friedman and Reeves show how contemporary elites tend to perform cultural ordinariness vis‐à‐vis the broader public and each other, we actually show that private museum founders actively mobilize their involvement in the arts to fashion a refined and culturally competent self‐image. We argue that this operates as a marker of distinction toward other segments of the economic elite, conveying not only cultural capital but also claims to moral worth (Bourdieu 1984; Kolbe 2023; Sherman 2018).

However, while our paper overall argues that private museums therefore serve their founders as productive sites of (self)legitimation claims, these are indeed only claims. The question of whether museum founders actually succeed in their legitimation work is beyond the scope of our study. Indeed, there is some evidence that shows how legitimation strategies around private museums may, in fact, not actually be effective, at least not within the artworld itself (Rozenbaum Friedman, forthcoming). How mission statement narratives interact with other legitimation efforts of museums and their founders, such as media interviews, board memberships, and biennial participation, to shape elite status in practice are therefore promising avenues for future research.

Lastly, while this paper focused on art philanthropy and on private museum institutions specifically, other philanthropic foundations such as the Bill & Melinda Gates Foundation, the Open Society Foundations, and the Mohammed bin Rashid Al Maktoum Knowledge Foundation employ remarkably similar narrative structures^5^, such as biographical family references, institutional timelines, and a focus on education and research. This raises the question of how fundamentally different private museums are from large foundations in terms of elite spending, influence‐seeking, and legitimation practices. More comparative studies on private philanthropy across different sectors would be needed to discern whether certain social domains lend themselves particularly well for contemporary elites in their pursuit to legitimize their resources, status, and influence. We would encourage future research on private museums to explore these issues through more localized case studies to get a better understanding of these dynamics and to potentially uncover additional, region‐specific legitimation work.

## Funding

Funding for this research was enabled by a grant from the Dutch Research Council (NWO) [Grant No. 406.18.CW.012] and forms part of the project ‘The Return of the Medici?’

## Figures and Tables

**FIGURE A1:**
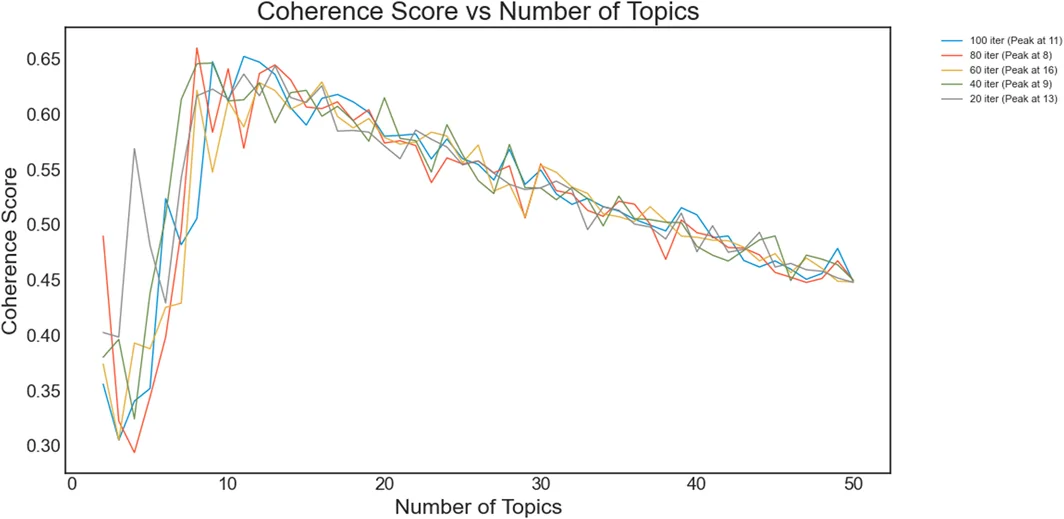
Coherence scores.

## Data Availability

Research data are not shared.

## Endnotes

^1^
In an interview with Financial Times, Bill Gates famously criticised his fellow billionaires who direct philanthropic activities toward the arts, for instance by financing a museum's new wing, rather than fund research which could eradicate illnesses that could make you blind: ‘The moral equivalent is, we're going to take 1 per cent of the people who visit this [museum] and blind them,’ he says. ‘Are they willing, because it has the new wing, to take that risk? Hmm, maybe this blinding thing is slightly barbaric’ (Waters 2013).

^2^
Founders' industry backgrounds were compiled and organized according to the highest level of aggregation of the International Standard Industrial Classification of All Economic Activities (ISIC) system.

^3^
The database also includes data which are beyond the scope of this paper, such as the collection focus, exhibition programming, public‐facing services (from opening days and ticketing through to further services such as museum café, shop, education or community programs), and digital presence (websites, official social media accounts).

^4^
Most mission statements were available in English; in case they were not, we translated them using Google Translate.

^5^
See ‘About’ section on Bill and Melinda Gates Foundation website. Available on: https://www.gatesfoundation.org/about/our‐story; ‘Who we are’ section on the Open Society Foundation website. Available on: https://www.opensocietyfoundations.org/who‐we‐are; ‘About us’ section on the Mohammed bin Rashid Al Maktoum Knowledge Foundation website. Available on: https://mbrf.ae/en/foundation/about‐us.

## Appendix

Figure A1

## References

[bjos70102-bib-0001] Accominotti, F. , S. Khan , and A. Storer . 2018. “How Cultural Capital Emerged in Gilded Age America: Musical Purification and Cross‐Class Inclusion at the New York Philharmonic.” American Journal of Sociology 123, no. 6: 1743–1783. 10.1086/696938.

[bjos70102-bib-0002] Adam, G. 2021. The Rise and Rise of the Private Art Museum, 104. Lund Humphries Publishers.

[bjos70102-bib-0003] Ajana, B. 2015. “Branding, Legitimation and the Power of Museums: The Case of the Louvre Abu Dhabi.” Museum & Society 13, no. 3: 322–341. 10.29311/mas.v13i3.333.

[bjos70102-bib-0103] Alegre, I. , J. Berbegal‐Mirabent , A. Guerrero , and M. Mas‐Machuca . 2018. “The Real Mission of the Mission Statement: A Systematic Review of the Literature.” Journal of Management and Organization 24, no. 4: 456–473. 10.1017/jmo.2017.82.

[bjos70102-bib-0104] Bechtler, C. , and D. Imhof . 2018. The Private Museum of the Future. JRP Ringier, Les Presses du reél.

[bjos70102-bib-0105] Bishop, C. 2012. Artificial Hells: Participatory Art and the Politics of Spectatorship. Verso.

[bjos70102-bib-0106] Bishop, C. 2025. “The Social Turn: Collaboration and its Discontents.” In The Performance Studies Reader, 250–257. Routledge.

[bjos70102-bib-0107] Bishop, C. , and D. Perjovschi . 2014. Radical Museology or, “what’s Contemporary” in Museums of Contemporary Art? 2nd ed. Koenig Books.

[bjos70102-bib-0108] Borgdorff, H. 2006. The Debate on Research in the Arts. 2nd ed. Kunsthøgskolen i Bergen Bergen.

[bjos70102-bib-0109] Borgdorff, H. 2010. “The Production of Knowledge in Artistic Research.” In The Routledge Companion to Research in the Arts, 44–63. Routledge. https://www.taylorfrancis.com/chapters/edit/10.4324/9780203841327‐4/production‐knowledge‐artistic‐research‐henk‐borgdorff.

[bjos70102-bib-0110] Bourriaud, N. 2002. Relational Aesthetics: [Dijon] : Les Presses du réel.

[bjos70102-bib-0004] Boucher, B. 2020. “Billionaire Sheldon Solow Allegedly Gamed the Tax System for Years With His Inaccessible Art Museum.” Now His Widow Will Open It to the Public.

[bjos70102-bib-0005] Bourdieu, P. 1984. Distinction: A Social Critique of the Judgement of Taste. Routledge.

[bjos70102-bib-0006] Bourdieu, P. 1985. “The Market of Symbolic Goods.” Poetics 14, no. 1–2: 13–44. 10.1016/0304-422X(85)90003-8.

[bjos70102-bib-0007] Bourdieu, P. 1993. The Field of Cultural Production : Essays on Art and Literature. Columbia University Press.

[bjos70102-bib-0111] Braden, L. E. A. 2016. “Collectors and Collections: Critical Recognition of the World’s Top Art Collectors.” Social Forces 94, no. 4: 1483–1507. 10.1093/sf/sov116.

[bjos70102-bib-0010] Brown, K. 2019. “Private Influence, Public Goods, and the Future of Art History.” Journal for Art Market Studies 3, no. 1. 10.23690/jams.v3i1.86.

[bjos70102-bib-0011] Brown, K. , and J. Pes . 2018. How Much Should Taxpayers Pay for a Private Museum? Artnet News.

[bjos70102-bib-0112] Brown, K. 2020. “When Museums Meet Markets.” Journal of Visual Art Practice 19, no. 3: 203–210. 10.1080/14702029.2020.1811488.

[bjos70102-bib-0012] Bryson, B. 1996. “Anything But Heavy Metal’: Symbolic Exclusion and Musical Dislikes.” American Sociological Review 61, no. 5: 884–899. 10.2307/2096459.

[bjos70102-bib-0113] Buchholz, L. 2022. “The Global Rules of Art.” In The Global Rules of Art. Princeton University Press.

[bjos70102-bib-0015] Castelló, I. , M. Etter , and F. Å. Nielsen . 2016. “Strategies of Legitimacy Through Social Media: The Networked Strategy.” Journal of Management Studies 53, no. 3: 402–432. 10.1111/joms.12145.

[bjos70102-bib-0016] Chen, Y. , H. Zhang , R. Liu , Z. Ye , and J. Lin . 2019. “Experimental Explorations on Short Text Topic Mining Between LDA and NMF Based Schemes.” Knowledge‐Based Systems 163: 1–13. 10.1016/j.knosys.2018.08.011.

[bjos70102-bib-0017] Cousin, B. , S. Khan , and A. Mears . 2018. “Theoretical and Methodological Pathways for Research on Elites.” Socio‐Economic Review 16, no. 2: 225–249. 10.1093/ser/mwy019.

[bjos70102-bib-0018] De Keere, K. , and L. Burchartz . 2025. “Entering Finance: Justifying Career Choices in a Stigmatized Industry.” American Journal of Cultural Sociology 13, no. 3: 1–36. 10.1057/s41290-024-00251-7.

[bjos70102-bib-0019] Dercon, C. 2017. “Why Bother? or the Rise of the Private Museum.” In Art Centre Basel.” in New Museums: Intentions, Expectations, Challenges; Musée d’Art Et d’Histoire, edited by K. Beisiegel and S. MacLeod . Hirmer.

[bjos70102-bib-0021] Dimaggio, P. 1982. “Cultural Entrepreneurship in Nineteenth‐Century Boston: The Creation of an Organizational Base for High Culture in America.” Media, Culture & Society 4, no. 1: 33–50. 10.1177/016344378200400104.

[bjos70102-bib-0022] DiMaggio, P. 1991. “Constructing an Organizational Field as a Professional Project: U.S. Art Museums, 1920‐1940.” In The New Institutionalism in Organizational Analysis, edited by P. DiMaggio and W. W. Powell , 262–297. University of Chicago Press.

[bjos70102-bib-0023] DiMaggio, P. , and W. W. Powell . 1983. “The Iron Cage Revisited: Institutional Isomorphism and Collective Rationality in Organizational Fields.” American Sociological Review 48, no. 2: 147–160. 10.2307/2095101.

[bjos70102-bib-0024] Egger, R. 2022. “Topic Modelling: Modelling Hidden Semantic Structures in Textual Data.” In Applied Data Science in Tourism, Tourism on the Verge, edited by R. Egger , 375–403. Springer International Publishing.

[bjos70102-bib-0025] Egger, R. , and J. Yu . 2022. “A Topic Modeling Comparison Between LDA, NMF, Top2Vec, and BERTopic to Demystify Twitter Posts.” Frontiers in Sociology 7: 886498. 10.3389/fsoc.2022.886498. 35602001 PMC9120935

[bjos70102-bib-0026] Farrell, J. 2020. Billionaire Wilderness: The Ultra‐Wealthy and the Remaking of the American West. Princeton University Press.

[bjos70102-bib-0028] Friedman, S. , and A. Reeves . 2020. “From Aristocratic to Ordinary: Shifting Modes of Elite Distinction.” American Sociological Review 85, no. 2: 323–350. 10.1177/0003122420912941.

[bjos70102-bib-0114] Foster, H. 2015. After the White Cube. London Review of Books. https://www.lrb.co.uk/the‐paper/v37/n06/hal‐foster/after‐the‐white‐cube.

[bjos70102-bib-0115] Harth, A. , and B. George . 2025. “Understanding Politicians Attitudes to Reusing Built Heritage: An Experimental and Behavioral Approach.” Journal of Cultural Heritage 76: 299–306. 10.1016/j.culher.2025.10.013.

[bjos70102-bib-0031] Hearn, A. , and S. Banet‐Weiser . 2020. “The Beguiling: Glamour In/As Platformed Cultural Production.” Social Media + Society 6, no. 1: 2056305119898779. 10.1177/2056305119898779.

[bjos70102-bib-0116] Heemskerk, E. M. , and F. W. Takes . 2015. “The Corporate Elite Community Structure of Global Capitalism.” New Political Economy 21, no. 1: 90–118. 10.1080/13563467.2015.1041483.

[bjos70102-bib-0117] Helmore, E. 2011. “Anger at Walmart Heiress’s $1.4Bn Gallery as Art Market Becomes Focus for Protests.” The Guardian. https://www.theguardian.com/business/2011/nov/20/walmart‐gallery‐crystal‐bridges‐museum.

[bjos70102-bib-0033] Higonnet, A. 1997. “Private Museums, Public Leadership: Isabella Steward Gardner and the Art of Cultural Authority.” In Cultural Leadership in America: Art Matronage and Patronage, edited by W. M. Corn , 79–92. Trustees of the Isabella Stewart Gardner Museum.

[bjos70102-bib-0034] Higonnet, A. 2009. A Museum of One’s Own: Private Collecting, Public Gift. Periscope.

[bjos70102-bib-0118] IRAPFM 2025. Decrease in Public Funding? A Worldwide Answer from Museums, 1–665. ICOM. https://icom.museum/wp‐content/uploads/2025/02/IRAPFM‐FINAL_7fev_2025‐1.pdf.

[bjos70102-bib-0036] Jung, Y. 2015. “Diversity Matters: Theoretical Understanding of and Suggestions for the Current Fundraising Practices of Nonprofit Art Museums.” Journal of Arts Management, Law, and Society 45, no. 4: 255–268. 10.1080/10632921.2015.1103672.

[bjos70102-bib-0037] Kantola, A. , and H. Kuusela . 2019. “Wealth Elite Moralities: Wealthy Entrepreneurs’ Moral Boundaries.” Sociology 53, no. 2: 368–384. 10.1177/0038038518768175.

[bjos70102-bib-0038] Khan, S. 2012. “The Sociology of Elites.” Annual Review of Sociology 38, no. 1: 361–377. 10.1146/annurev-soc-071811-145542.

[bjos70102-bib-0039] Kolbe, K. J. 2023. “The Art of (Self) Legitimization: How Private Museums Help Their Founders Claim Legitimacy as Elite Actors.” Socio‐Economic Review.

[bjos70102-bib-0119] Lamont, M. , and L. Thévenot . 2000. “Rethinking Comparative Cultural Sociology.” Repertoires of Evaluation 8.

[bjos70102-bib-0040] Lamont, M. , S. Beljean , and M. Clair . 2014. “What is Missing? Cultural Processes and Causal Pathways to Inequality.” Socio‐Economic Review 12, no. 3: 573–608. 10.1093/ser/mwu011.

[bjos70102-bib-0041] Larry’s List . 2016. “Private Art Museum Report.”. Art Market Monitor of Artron, edited by Bouchara, C. , M. Bossier , C. Howald , S. Liu , and C. Noe . Modern Arts Publishing.

[bjos70102-bib-0042] Larsen, H. 2017. “10 the Public Sphere as an Arena for Legitimation Work: The Case of Cultural Organizations.” In Institutional Change in the Public Sphere, edited by F. Engelstad , H. Larsen , J. Rogstad , and K. Steen‐Johnsen , 201–219. De Gruyter Open.

[bjos70102-bib-0043] Lena, J. C. 2019. Entitled: Discriminating Tastes and the Expansion of the Arts. Princeton University Press.

[bjos70102-bib-0044] Maclean, M. , C. Harvey , and R. Chia . 2012. “Sensemaking, Storytelling and the Legitimization of Elite Business Careers.” Human Relations 65, no. 1: 17–40. 10.1177/0018726711425616.

[bjos70102-bib-0045] Maclean, M. , C. Harvey , R. Yang , and F. Mueller . 2021. “Elite Philanthropy in the United States and United Kingdom in the New Age of Inequalities.” International Journal of Management Reviews 23, no. 3: 1–23. 10.1111/ijmr.12247.

[bjos70102-bib-0046] McGoey, L. 2012. “Philanthrocapitalism and Its Critics.” Poetics 40, no. 2: 185–199. 10.1016/j.poetic.2012.02.006.

[bjos70102-bib-0047] Mears, A. 2020. Very Important People: Status and Beauty in the Global Party Circuit. Princeton University Press.

[bjos70102-bib-0120] Mendoza, H. M. , and A. S. Talavera . 2025. “Governance Strategies for the Management of Museums and Heritage Institutions.” Heritage 8, no. 4: 127. 10.3390/heritage8040127.

[bjos70102-bib-0048] Milner, A. , and C. Hartnell . 2015. ““An Act of Faith? Why Should Philanthropists Fund the Arts?” Alliance Magazine.” (March). https://www.alliancemagazine.org/feature/an‐act‐of‐faith‐why‐should‐philanthropists‐fund‐the‐arts/.

[bjos70102-bib-0050] Moulin, R. 1987. The French Art Market: A Sociological View. Rutgers University Press.

[bjos70102-bib-0121] Pabst, K. 2016. Towards New Relations Between the Museum and Society (ICOM Norwegian National Committee (ICOM Norway). Vest‐Agder‐museene.

[bjos70102-bib-0051] Peterson, R. A. , and R. M. Kern . 1996. “Changing Highbrow Taste: From Snob to Omnivore.” American Sociological Review 61, no. 5: 900–907. 10.2307/2096460.

[bjos70102-bib-0052] Piketty, T. 2014. Capital in the Twenty‐First Century. Harvard University Press.

[bjos70102-bib-0053] Piketty, T. 2021. “Capital and Ideology.” Journal of Social Policy 50, no. 2: 440–441. 10.1017/S0047279421000039.

[bjos70102-bib-0054] Prieur, A. , and M. Savage . 2013. “Emerging Forms of Cultural Capital.” European Societies 15, no. 2: 246–267. 10.1080/14616696.2012.748930.

[bjos70102-bib-0055] Reeves, A. , and S. Friedman . 2024. Born to Rule: The Making and Remaking of the British Elite. Harvard University Press.

[bjos70102-bib-0056] Reich, R. 2018. Just Giving: Why Philanthropy is Failing Democracy and How it Can Do Better. Princeton University Press.

[bjos70102-bib-0057] Robson, D. 2019. “A Law Of Unintended Consequences? United States Federal Taxation and the Market for Modern Art in the United States.” Journal for Art Market Studies 3, no. 1. 10.23690/jams.v3i1.69.

[bjos70102-bib-0122] Röder, M. , A. Both , and A. Hinneburg . 2015. “Exploring the Space of Topic Coherence Measures.” Proceedings of the Eighth ACM International Conference on Web Search and Data Mining, 399–408. 10.1145/2684822.2685324.

[bjos70102-bib-0058] Rogers, T. 2022. “Artists Scrutinize Nazi Family Past of Julia Stoschek.” New York Times, (June).

[bjos70102-bib-0059] Rothkopf, D. 2008. Superclass: The Global Power Elite and the World They Are Making. Farrar, Straus and Giroux.

[bjos70102-bib-0060] Rushing, D. 2022. The Art World is a Haven for the Superrich. Jacobin.

[bjos70102-bib-0061] Savage, M. , and J. Hjellbrekke . 2021. The Sociology of Elites: A European Stocktaking and Call for Collaboration. Working Paper 58, 1–47. LSE International Inequalities Institute.

[bjos70102-bib-0123] Saunders‐Hastings, E. 2024. Private Virtues, Public Vices: Philanthropy and Democratic Equality. University of Chicago Press.

[bjos70102-bib-0124] Schmitz, H. P. , G. E. Mitchell , and E. M. McCollim . 2021. “How Billionaires Explain Their Philanthropy: A Mixed‐Method Analysis of the Giving Pledge Letters.” Voluntas: International Journal of Voluntary and Nonprofit Organizations 32, no. 2: 512–523. 10.1007/s11266-021-00338-6.

[bjos70102-bib-0125] Shaked, N. 2022. Museums and Wealth: The Politics of Contemporary Art Collections. Bloomsbury Academic.

[bjos70102-bib-0062] Sherman, R. 2018. “A Very Expensive Ordinary Life’: Consumption, Symbolic Boundaries and Moral Legitimacy Among New York Elites.” Socio‐Economic Review 16, no. 2: 411–433. 10.1093/ser/mwy011.

[bjos70102-bib-0126] Spence, R. 2024. Battle for the Museum: Cultural Institutions in Crisis. Oxford University Press.

[bjos70102-bib-0127] Suchman, M. C. 1995. “Managing Legitimacy: Strategic and Institutional Approaches.” Academy of Management Review 20, no. 3: 571. 10.2307/258788.

[bjos70102-bib-0128] Toepler, S. 2022. “Legitimizing Foundations: Roles, Expectations, and Regulation.” In Civil Society: Concepts, Challenges, Contexts, edited by M. Hoelscher , R. A. List , A. Ruser , and S. Toepler , 151–166. Springer International Publishing. 10.1007/978-3-030-98008-5_10.

[bjos70102-bib-0064] Velthuis, O. , and M. Gera . 2024. “The Fragility of Cultural Philanthropy: Why Private Art Museums Close.” International Journal of Cultural Policy 31, no. 6: 1–14. 10.1080/10286632.2024.2365281.

[bjos70102-bib-0065] Velthuis, O. , K. Kolbe , J. Aengenheyster , A. Friedmann Rozenbaum , M. Gera , and M. Zhang . 2023. Beyond the Global Boom: Private Art Museums in the 21st Century.

[bjos70102-bib-0066] Walker, G. S. 2019. The Private Collector’s Museum: Public Good Versus Private Gain. Routledge.

[bjos70102-bib-0068] Waters, R. 2013. An Exclusive Interview with Bill Gates. Financial Times.

[bjos70102-bib-0129] Worth, E. , A. Reeves , and S. Friedman . 2023. “Is There an Old Girls' Network? Girls' Schools and Recruitment to the British Elite.” British Journal of Sociology of Education 44, no. 1: 1–25. 10.1080/01425692.2022.2132472.

[bjos70102-bib-0072] Zolberg, V. 1984. “Conflicting Visions in American Art Museums.” Social Forces 63, no. 2: 377–392. 10.2307/2579052.

[bjos70102-bib-0073] Zolberg, V. 2000. “Privatization: Threat or Promise for the Arts and Humanities?” International Journal of Cultural Policy 7, no. 1: 9–27. 10.1080/10286630009358130.

